# Testosterone Upregulates the Expression of Mitochondrial ND1 and ND4 and Alleviates the Oxidative Damage to the Nigrostriatal Dopaminergic System in Orchiectomized Rats

**DOI:** 10.1155/2017/1202459

**Published:** 2017-08-24

**Authors:** Wensheng Yan, Yunxiao Kang, Xiaoming Ji, Shuangcheng Li, Yingkun Li, Guoliang Zhang, Huixian Cui, Geming Shi

**Affiliations:** ^1^Department of Neurobiology, Hebei Medical University, Shijiazhuang, Hebei 050017, China; ^2^Department of Human Anatomy, Shijiazhuang Medical College, Shijiazhuang, Hebei 050599, China; ^3^Department of Human Anatomy, Hebei Medical University, Shijiazhuang, Hebei 050017, China

## Abstract

Testosterone deficiency, as a potential risk factor for aging and aging-related neurodegenerative disorders, might induce mitochondrial dysfunction and facilitate the declines of the nigrostriatal dopaminergic system by exacerbating the mitochondrial defects and increasing the oxidative damage. Thus, how testosterone levels influence the mitochondrial function in the substantia nigra was investigated in the study. The present studies showed that testosterone deficiency impaired the mitochondrial function in the substantia nigra and induced the oxidative damage to the substantia nigra as well as the deficits in the nigrostriatal dopaminergic system. Of four mitochondrial respiratory chain complexes, castration of male rats reduced the activity of mitochondrial complex I and downregulated the expression of ND1 and ND4 of 7 mitochondrial DNA- (mtDNA-) encoded subunits of complex I in the substantia nigra. Supplements of testosterone propionate to castrated male rats ameliorated the activity of mitochondrial complex I and upregulated the expression of mitochondrial ND1 and ND4. These results suggest an important role of testosterone in maintaining the mitochondrial function in the substantia nigra and the vulnerability of mitochondrial complex I to testosterone deficiency. Mitochondrial ND1 and ND4, as potential testosterone targets, were implicated in the oxidative damage to the nigrostriatal dopaminergic system.

## 1. Introduction

Oxidative stress plays a key role in aging and aging-related neurodegenerative disorders [1–3], such as Parkinson's disease (PD). Testosterone deficiency, as a potential risk factor for neurodegenerative disorders [4], is implicated in oxidative stress [5–8]. Orchiectomy elevates the susceptibility of brain tissue to oxidative stress [6, 7]. Oxidative stress-mediated damage to neurons can be manipulated by testosterone administration [5–8]. Testosterone supplements reduce the oxidative damage by increasing antioxidant enzyme levels [6] and ameliorating the oxidative stress parameters [8, 9] in brain tissues. In vitro studies reveal that the cerebellar granule cells from neonatal rats treated with testosterone are selectively protected against oxidative stress-induced cell death [5]. Testosterone is involved in the protection of neurons via suppressing oxidative stress.

Normal neuronal activities are critically dependent on mitochondrial function [10]. Mitochondria, as primary sources of reactive oxygen species (ROS) and primary targets of ROS damage [1, 2, 11–13], have been proposed to play an important role in the pathogenesis of neurodegenerative disorders [1, 2, 14, 15]. The defects of mitochondria, such as the reduced activity of the mitochondrial respiratory chain and the overproduced ROS, are detected in the brains of subjects with aging-related neurodegenerative disorders [16–21]. Mitochondrial dysfunction induces a progressive disruption of the redox balance and is implicated in aging or aging-related neurodegeneration.

In normal aging, the nigrostriatal dopaminergic system progressively declines [22–25], with a decrease in the number of dopaminergic neurons [22, 23] and dopamine (DA) content [24, 25]. Although several factors have been proposed for the declined dopaminergic system in the aging process, one of the major contributors is oxidative stress [26–28]. PD, as a common neurodegenerative movement disorder, pathologically undergoes neurodegenerative loss of dopamine neurons in the substantia nigra [29]. Age-related mitochondrial alterations are demonstrated in the human skeletal muscle beginning at 40 ~ 50 years of age [30, 31]. Coincidentally, changes in the sexual hormonal state of individuals also start at this age interval [32], which suggests a relationship between hormonal levels and mitochondrial status [33]. With advancing age, the reduced levels of testosterone in aged males [34–37] might facilitate the declines of the nigrostriatal dopaminergic system by exacerbating the mitochondrial defects [15, 38] and increasing the oxidative damage in the substantia nigra.

Based on the effects of testosterone on oxidative stress-mediated damage to neurons [5–9], the association of mitochondria with oxidative stress [11, 12], and the amelioratory effects of testosterone on the deficits in the nigrostriatal dopaminergic system of aged male rats [9, 39, 40], we presumed that the amelioratory effects of testosterone on the impaired nigrostriatal dopaminergic system might be realized by regulating the function of mitochondria in a way. Testosterone deficiency might intervene the mitochondrial function in the substantia nigra. Therefore, in the present study, the dopaminergic markers in the nigrostriatal dopaminergic system and the parameters related to mitochondria were analyzed in male rats by manipulating serum testosterone levels to testify which of mitochondrial DNA- (mtDNA-) encoded subunits, as potential testosterone targets, was implicated in the substantia nigra.

## 2. Materials and Methods

### 2.1. Animals

Adult male Sprague–Dawley rats (280–300 g) were supplied by the Experimental Animal Center of Hebei Medical University. All of the rats were kept in groups of four per cage and housed under controlled temperature (22 ± 1°C) and humidity conditions with a 12 h light-dark cycle (lights on 06:00 h). Food and water were available ad libitum. The experimental procedures followed the rules in the “Guidelines for the Care and Use of Mammals in Neuroscience and Behavioral Research” and were approved by the Committee of Institutional Animal Care and Use of Hebei Medical University.

### 2.2. Treatments

For testosterone deficiency, the rats anesthetized with chloral-hydrate (300 mg/kg) were gonadectomized (GDX) by the removal of the testes, epididymis, and epididymal fat under aseptic conditions, and the incisions were closed using surgical staples. The sham-operated rats experienced the same surgical treatment except for the bilateral orchiectomies (sham) [41]. For testosterone replacement, testosterone propionate (TP) was injected subcutaneously to GDX rats based on the following experimental schemes. The GDX rats receiving sesame oil treatment were used as a control.

### 2.3. Designs

#### 2.3.1. Experiment 1

Eighty rats were used to determine the optimal dose of testosterone replacement by detecting the levels of malondialdehyde (MDA), hydrogen peroxide (H_2_O_2_), reduced glutathione (GSH), and oxidized glutathione (GSSG) in the substantia nigra (SN). They were included in the following groups: sham (*n* = 8), GDX (*n* = 8), or GDX-TP (*n* = 64). Eight rats in GDX-TP received 28-day treatment of TP at 0.5, 0.75, 1.0, 1.25, 1.5, 2.0, 2.5, or 3.0 mg/kg.

#### 2.3.2. Experiment 2

Sixty-four rats were used to investigate the effect of testosterone deficiency and testosterone replacement on the nigrostriatal dopaminergic system. They were included in the following four groups: sham (*n* = 16), GDX (*n* = 16), GDX-0.5TP (*n* = 16), or GDX-1.0TP (*n* = 16). Eight rats in each group were used for real-time quantitative PCR (qPCR) and liquid chromatography coupled with tandem mass spectrometry (LC-MS/MS) assay or for Western blot detection. The rats in GDX-0.5TP and GDX-1.0TP experienced 28-day treatment of TP at 0.5 mg/kg and 1.0 mg/kg, respectively. Two TP doses were chosen based on the results in the experiment 1.

#### 2.3.3. Experiment 3

One hundred and twenty-eight rats were used to investigate the effect of testosterone deficiency and testosterone replacement on mitochondrial function. They were included in the following four groups: sham (*n* = 32), GDX (*n* = 32), GDX-0.5TP (*n* = 32), or GDX-1.0TP (*n* = 32). Eight rats in each group were used for detection of the mitochondrial membrane potential and the activity of mitochondrial complexes, qPCR, or Western blot detection. The rats in GDX-0.5TP and GDX-1.0TP experienced 28-day treatment of TP at 0.5 mg/kg and 1.0 mg/kg, respectively. TP dose was chosen based on the results in experiment 1.

### 2.4. Sample Preparation

The rats were sacrificed by decapitation. The tissue block containing the SN (between 3.00 mm and 4.08 mm rostral to the interaural axis) or the caudate-putamen (CPu; between 10.08 mm and 8.64 mm rostral to the interaural axis) [42] was dissected on an ice-cold plate, using a scalpel for ophthalmic surgery and stereomicroscopy. It was immediately processed for assays of MDA, H_2_O_2_, GSH/GSSG, and mitochondrial membrane potential as well as the activity of mitochondrial complexes or stored at −80°C after being frozen in liquid nitrogen until further qPCR, Western blot, or LC-MS/MS assay based on the experiment purposes.

### 2.5. Mitochondrial Membrane Potential

The mitochondrial membrane potential in the SN was detected using the rhodamine 123 (Rh123) fluorescence method [10]. The pieces containing the SN were grinded with a balanced salt solution and filtered through a nylon mesh screen. The cells were collected and incubated in Rh123 solution (10 *μ*g/mL, Sigma, USA) at 37°C for 30 min. After being washed and resuspended in 1 mL PBS, the cells were immediately analyzed by flow cytometry (excitation/emission wavelengths, 488/534 nm). The mitochondrial membrane potential was assessed by the change in the intensity of Rh123 fluorescence. Weak fluorescent intensity of Rh123 reflects the decreased mitochondrial membrane potential.

### 2.6. Biochemical Analysis

For MDA assay, SN tissue block was homogenized with 10 times (*w*/*v*) ice-cold 0.1 M phosphate buffer (PB) at pH 7.4. The homogenate was then centrifuged at 14,000*g* for 15 min at 4°C followed by recovery of the supernatant. The supernatant of SN homogenates was used to detect MDA. The levels of MDA were measured spectrophotometrically according to the protocol of the detection kits. The detection kit (Code A003-1) was obtained from the Jiancheng Institute of Biotechnology of China.

For the detection of H_2_O_2_ and GSH/GSSG in mitochondria of the SN, the mitochondria were isolated using the Tissue Mitochondria Isolation Kit (Code C3606, Beyotime Institute of Biotechnology, China). In brief, SN tissue was homogenized in ice-cold buffer (10 mM HEPES, pH 7.5, including 200 mM mannitol, 70 mM sucrose, 1.0 mM EGTA, and 2.0 mg/mL serum albumin) and centrifuged at 1000*g* at 4°C for 10 min. The supernatant was centrifuged again at 3500*g* at 4°C for 10 min to collect a mitochondrial pellet. The levels of H_2_O_2,_ GSH, and GSSG in the mitochondria were measured spectrophotometrically according to the protocol of the detection kits (H_2_O_2_: Code A064-1, GSH: Code A006-1, and GSSG: Code A061-2; Jiancheng Institute of Biotechnology, China).

For the activity of mitochondrial complexes, isolated mitochondria were detected by spectrophotometric assays to reveal the complex activity of the mitochondrial electron transfer chain. The activity of complexes I, II, III, and IV was measured according to the protocol of the detection kits. The detection kits for complex I (Code S50007), complex II (Code S50008), complex III (Code S50009), and complex IV (Code S50010) were obtained from the Nanjing Jiancheng Institute of Biotechnology of China.

### 2.7. LC-MS/MS Assay

CPu tissue block in experiment 2 was weighed and homogenized in 80% acetonitrile containing 0.1% formic acid (5 *μ*L). The homogenates were centrifuged at 14,000*g* for 10 min at 4°C. The supernatants were collected and stored at −80°C. DA, 3,4-dihydroxyphenylacetic acid (DOPAC), and homovanillic acid (HVA) were determined by the use of LC-MS/MS. LC separation was performed on the Agilent 1200 LC system (Agilent, Santa Clara, USA) using a Synergi Fusion-RP C18 column (50 mm × 3.0 mm, 4 *μ*m) provided by Phenomenex. MS/MS detection was carried out using the 3200 QTRAP LC-MS/MS System (Applied Biosystems, Foster City, CA, USA). The multiple-reaction monitoring mode was used for the quantification. The principal validation parameters of the LC-MS/MS were set up as showed in Table 1.

### 2.8. qPCR Analysis

2 *μ*g of total RNA from SN tissue block was subjected to reverse transcription using random primers to obtain the first-strand cDNA template. qPCR was performed with 0.8 *μ*L cDNA (diluted 1 : 10), 2 *μ*L specific primers, and 2× GoTaq® Green Master Mix (Promega, USA) with a final volume of 20 *μ*L. PCR was performed as follows: an initial cycle at 95°C for 10 min, followed by 40 cycles at 95°C for 15 s, 58°C for 20 s, and 60°C for 15 s. Then, PCR products were analyzed by the melting curve to confirm the specificity of amplification. Expression of tyrosine hydroxylase (TH) and dopamine transporter (DAT) genes as well as mtDNA-encoded complex I genes was analyzed using GAPDH or *β*-actin as an internal control. The relative quantification was calculated using the 2^−ΔΔct^ method. The sets of primers were as follows—TH: (forward) 5′-GCTTCTCTGACCAGGTGTATCG-3′ and (reverse) 5′-GCAATCTCTTCCGCTGTGTAT-3′, DAT: (forward) 5′-ACTCTGTGAGGCATCTGTGTG-3′ and (reverse) 5′-TGTAACTGGAGAAGGCAATCAG-3′, GAPDH: (forward) 5′-TGAACGGGAAGCTCACTG-3′ and (reverse) 5′-GCTTCACCACCTTCTTGATG-3′, ND1: (forward) 5′-CCTATGAATCCGAGCATCC-3′ and (reverse) 5′-ATTGCAGGGAAATGTATCA-3′, ND2: (forward) 5′-CAACCAACAACAACTCCAAA-3′ and (reverse) 5′-AAAGCGGTAGGGTAAGGGTA-3′, ND3: (forward) 5′-AGTTCTGCACGCCTTCCTT-3′ and (reverse) 5′-ATCCACACAGATGCCTCACA-3′, ND4: (forward) 5′-CCCTACCCTCAACATGATCC-3′ and (reverse) 5′-GGAGCTTCTACGTGGGCTTT-3′, ND4L: (forward) 5′-TCCACATTAAACTCCAACTCCA-3′ and (reverse) 5′-CGTAGTCTGTTCCGTAAGTATTTGA-3′, ND5: (forward) 5′-CCAACCCTACCTTGCTTTCC-3′ and (reverse) 5′-GGCTCCCGATAATGAGACAA-3′, ND6: (forward) 5′-GTCTCCGGGTACTCCTCAGT-3′ and (reverse) 5′-GTGGGCTTGGATTGATTGTT-3′, and *β*-actin: (forward) 5′-TCATGAAGTGTGACGTTGACATCCGT-3′ and (reverse) 5′-CCTAGAAGCATTTGCGGTGCACGATG-3′.

### 2.9. Western Blot Analysis

The tissue block for Western blot was homogenized in radioimmunoprecipitation assay (RIPA) buffer containing 1% Triton X-100, 0.1% SDS, 0.5% sodium deoxycholate, and protease inhibitors (100 *μ*g/mL phenylmethanesulfonyl fluoride, 30 *μ*g/mL aprotinin, and 1 mM sodium orthovanadate) and then sonicated for 4 × 10 s. After centrifugation at 12,000*g* for 20 min at 4°C, the supernatant was collected and centrifuged again as above. The final resulting supernatant was stored at −80°C until use. Samples from the SN or CPu were diluted in 2× sample buffer (50 mM Tris (pH 6.8), 2% SDS, 10% glycerol, 0.1% bromophenol blue, and 5% *β*-mercaptoethanol), heated for 5 min at 95°C before SDS-PAGE on a 10% gel, and subsequently transferred to a PVDF membrane (Millipore). The membrane was incubated for 2 h with 5% nonfat dry milk in Tris-buffered saline (TBS) containing 0.05% Tween-20 (TBST). The membrane was rinsed in three changes of TBST and then incubated overnight with mouse anti-TH monoclonal antibody (1 : 10,000; T2928, Sigma), rabbit anti-DAT polyclonal antibody (1 : 4000; AB2231, Millipore), rabbit anti-ND1 monoclonal antibody (1 : 2000; ab181848, Abcam), or rabbit anti-ND4 monoclonal antibody (1 : 200; HPA053928, Sigma) at 4°C according to the experiment purposes. After three washes, the membrane was incubated for 1 h with IRDye® 800-conjugated goat anti-mouse second antibody (1 : 3000; Rockland) or goat anti-rabbit second antibody (1 : 5000; Rockland). The bands were scanned by an Odyssey infrared scanner (LI-COR Biosciences). Following stripping, each PVDF membrane was subsequently immunoblotted with mouse anti-*β*-actin monoclonal antibody (Santa Cruz Biotechnology). The labeling densities for TH, DAT, ND1, or ND4 were compared with those for *β*-actin, which were the endogenous control.

### 2.10. Testosterone Measurement

Trunk blood was collected from the rats after decapitation in experiment 1. Serum samples were prepared by centrifugation at 3000*g* for 15 min at 4°C and stored at −80°C until assay. Testosterone levels in the serum and in the SN supernatant of experiment 1 were measured by radioimmunoassay using the testosterone radioimmunoassay kit (Tianjin Nine Tripods Medical and Bioengineering Co. Ltd., China) in accordance with the manufacturer's protocol.

### 2.11. Statistical Analysis

All of the data are presented as the mean ± SD. We applied tests of normality (Kolmogorov-Smirnov test) and homogeneity variance (Levene's test). If both normal distribution (*P* > 0.1) and homogeneity of variance (*P* > 0.1) were found, then, parametric testing was performed by one-way analysis of variance (one-way ANOVA) followed by a Student-Newman-Keuls post hoc test for multiple comparisons. Otherwise, nonparametric statistics were done by the Kruskal-Wallis test followed by the Mann–Whitney *U* test for post hoc analysis between groups. The level of significance was taken as *P* < 0.05.

## 3. Results

### 3.1. Experiment 1

To choose the optimal doses of TP supplements in ameliorating the oxidative damage to the SN of GDX rats, MDA, mitochondrial H_2_O_2_, and mitochondrial GSH/GSSG were tested in rats by manipulating testosterone levels. Furthermore, the testosterone levels in serum and the SN were measured in experimental rats.

#### 3.1.1. MDA

**Figure 1:**
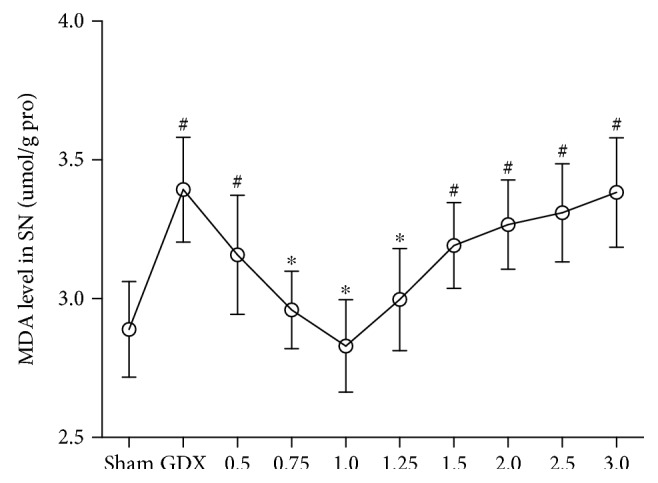
The level of MDA in the SN was dependent on the doses of TP supplements to GDX rats. *n* = 8 per group, expressed as mean ± SD. ^#^*P* < 0.01 versus sham rats, ^∗^*P* < 0.01 versus GDX rats.

Group differences were found in the level of MDA (Figure 1; one-way ANOVA, F(9,70) = 10.250, *P* < 0.01) among all groups. The post hoc test revealed that the level of MDA in the SN was significantly higher in GDX rats than in sham rats (*P* < 0.01). Supplements of TP at the dose of 0.75, 1.0, or 1.25 mg/kg reduced MDA in GDX rats to the level of the sham rats. TP supplements at a dose of over 1.5 mg/kg to GDX rats increased the level of MDA compared with the level of MDA of sham rats (*P* < 0.01).

#### 3.1.2. H_2_O_2_ and GSH/GSSG

**Figure 2:**
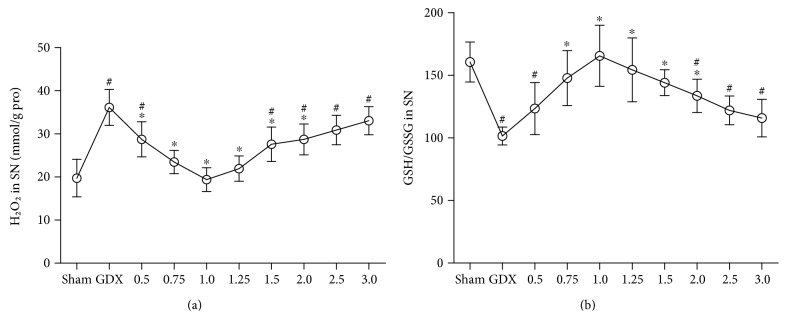
The mitochondrial H_2_O_2_ and GSH/GSSG in the SN were dependent on the doses of TP supplements to GDX rats. (a) Mitochondrial H_2_O_2_. (b) Mitochondrial GSH/GSSG. *n* = 8 per group, expressed as mean ± SD. ^#^*P* < 0.01 versus sham rats, ^∗^*P* < 0.01 versus GDX rats.

There were group differences in mitochondrial H_2_O_2_ (Figure 2(a); Kruskal-Wallis test, *χ*^2^ = 59.609, *P* < 0.01) and in mitochondrial GSH/GSSG (Figure 2(b); one-way ANOVA, F(9,70) = 11.340, *P* < 0.01) among all groups. The post hoc test showed the increased mitochondrial H_2_O_2_ production as well as the decreased mitochondrial GSH/GSSG in the SN of GDX rats compared with sham rats (*P* < 0.01). Increased mitochondrial H_2_O_2_ in GDX rats was restored to the levels in sham rats via TP supplement to GDX rats at the dose of 0.75, 1.0, or 1.25 mg/kg. The supplements of TP to GDX rats at the dose of 0.75, 1.0, 1.25, or 1.5 mg/kg brought the decreased GSH/GSSG to sham level.

#### 3.1.3. Testosterone Levels

**Figure 3:**
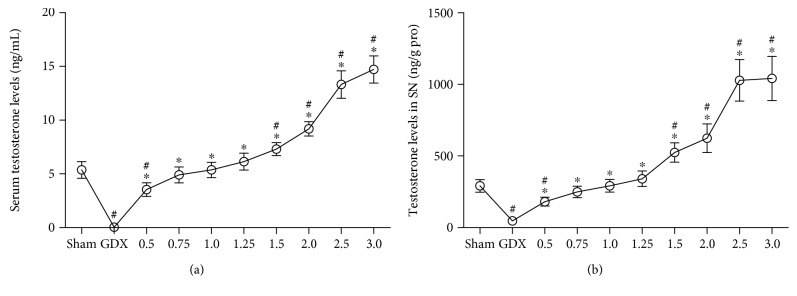
The testosterone levels in serum and in the SN were dependent on the doses of TP supplements to GDX rats. (a) Serum. (b) SN. *n* = 8 per group, expressed as mean ± SD. ^#^*P* < 0.01 versus sham rats, ^∗^*P* < 0.01 versus GDX rats.

Group differences among sham, GDX, and GDX-TP rats were found in the testosterone levels of serum (Figure 3(a); Kruskal-Wallis test, *χ*^2^ = 73.342, *P* < 0.01) and the SN (Figure 3(b); Kruskal-Wallis test, *χ*^2^ = 74.194, *P* < 0.01). GDX rats had the lowest serum and SN testosterone levels, which were hardly detected. Serum and SN testosterone levels in GDX rats that received the supplements of TP at 0.75, 1.0, or 1.25 mg/kg were in the same levels as those in sham rats. Supplements of TP at a dose of over 1.5 mg/kg to GDX rats resulted in supraphysiological levels of serum and SN testosterone (*P* < 0.01).

### 3.2. Experiment 2

The markers of the nigrostriatal dopaminergic system were analyzed to investigate the deficits in the nigrostriatal dopaminergic system and the effects of TP supplements on the impaired nigrostriatal dopaminergic system in GDX rats.

#### 3.2.1. DA and Its Metabolites

**Figure 4:**
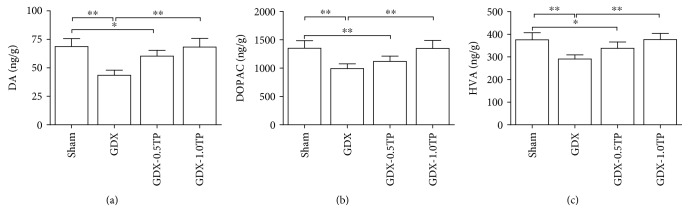
The effects of TP supplements on DA and its metabolites in the CPu of GDX rats. (a) DA. (b) DOPAC. (c) HVA. *n* = 8 per group, expressed as mean ± SD. ^∗^*P* < 0.05; ^∗∗^*P* < 0.01.

There were group differences in the levels of DA (Figure 4(a); one-way ANOVA, F(3, 28) = 29.021, *P* < 0.01), DOPAC (Figure 4(b); one-way ANOVA, F(3, 28) = 18.676, *P* < 0.01), and HVA (Figure 4(c), one-way ANOVA, F(3, 28) = 18.004, *P* < 0.01) in the CPu among sham, GDX, GDX-0.5TP, and GDX-1.0TP rats. The levels of DA, DOPAC, and HVA were decreased in GDX rats over sham rats by 36.68%, 26.63%, and 22.62% (*P* < 0.01), respectively. TP supplements to GDX rats at 1.0 mg/kg reversed them to the levels in sham rats. Supplements of TP at 0.5 mg/kg did not restore them to sham levels.

#### 3.2.2. TH and DAT

**Figure 5:**
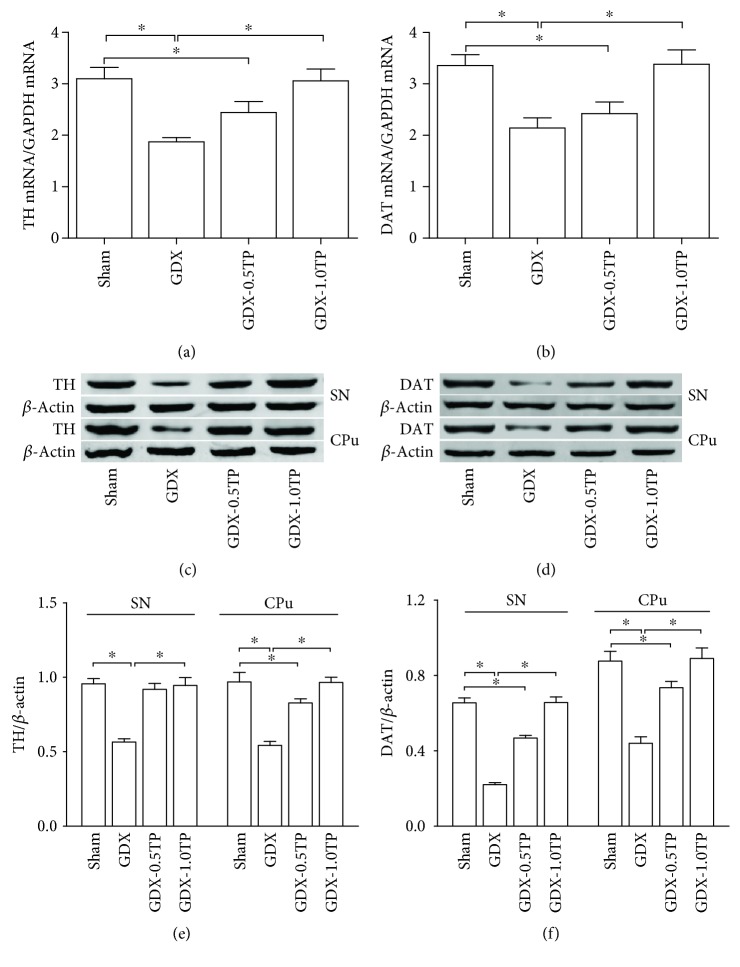
The effects of TP supplements on TH and DAT in GDX rats. (a-b) TH or DAT mRNA in the SN was detected by qPCR. (c–f) TH or DAT protein in the SN and CPu was measured by Western blot. *n* = 8 per group, expressed as mean ± SD. ^∗^*P* < 0.01.

Group differences were found in TH and DAT at the level of mRNA in the SN (Figure 5(a); TH: Kruskal-Wallis test, *χ*^2^ = 26.208, *P* < 0.01; Figure 5(b); DAT: one-way ANOVA, F(3, 28) = 61.358, *P* < 0.01) and at the level of protein both in the SN (Figures 5(c) and 5(e); TH: Kruskal-Wallis test, *χ*^2^ = 18.872, *P* < 0.01; Figures 5(d) and 5(f); DAT: Kruskal-Wallis test, *χ*^2^ = 26.189, *P* < 0.01) and in the CPu (Figures 5(c) and 5(e); TH: Kruskal-Wallis test, *χ*^2^ = 26.253, *P* < 0.01; Figures 5(d) and 5(f); DAT: one-way ANOVA, F(3, 28) = 174.783, *P* < 0.01) among sham, GDX, GDX-0.5TP, and GDX-1.0TP rats. The post hoc test showed that the TH and DAT mRNAs in the SN were, respectively, decreased by 39.68% and 36.31% in GDX rats over sham rats (*P* < 0.01). A 40.92% and 44.04% reduction in TH proteins, respectively, in the SN and in the CPu and a 66.41% and 49.78% decrease in DAT proteins were found in GDX rats compared with sham rats (*P* < 0.01). The significantly reduced TH and DAT at mRNA and protein levels in GDX rats were restored to the level in sham rats by the supplements of TP at 1.0 mg/kg, not at 0.5 mg/kg except for the protein level of TH in the SN.

### 3.3. Experiment 3

To determine the effects of testosterone on mitochondria in the SN, the parameters related to mitochondria were analyzed in male rats by manipulating serum testosterone levels and the implicated mtDNA-encoded subunits were identified in the substantia nigra of GDX rats.

#### 3.3.1. Mitochondrial Membrane Potential

**Figure 6:**
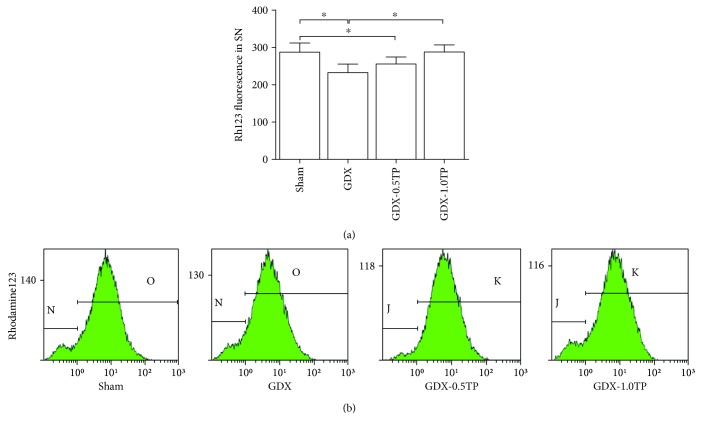
The effects of TP supplements on the mitochondrial membrane potential in the SN of GDX rats. *n* = 8 per group, expressed as mean ± SD. ^∗^*P* < 0.01.

Group differences of the Rh123 fluorescence intensity in the SN were found among sham, GDX, GDX-0.5TP, and GDX-1.0TP rats (Figure 6; one-way ANOVA, F(3,28) = 12.497, *P* < 0.01). The value of Rh123 fluorescence intensity was significantly lower in GDX rats than in sham rats by 19.12% (*P* < 0.01). Supplements of TP at 1.0 mg/kg, not at 0.5 mg/kg, restored the value of Rh123 fluorescence intensity in the SN of GDX rats to the level in sham rats.

#### 3.3.2. Mitochondrial Complex Activities

**Figure 7:**
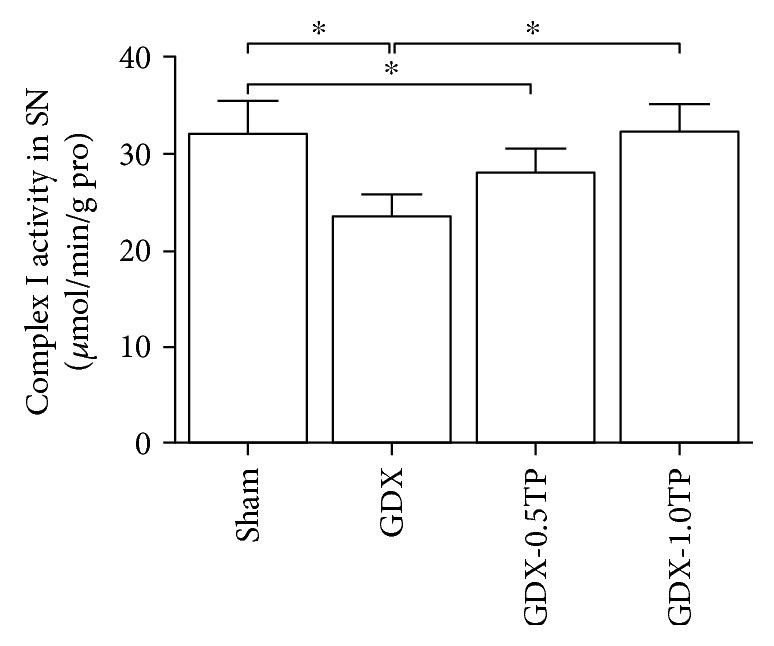
The effects of TP supplements on the activity of mitochondrial complex I in the SN of GDX rats. *n* = 8 per group, expressed as mean ± SD. ^∗^*P* < 0.01.

Of the four complexes of the mitochondrial respiratory chain, complex I in the SN showed a significant group difference among sham, GDX, GDX-0.5TP, and GDX-1.0TP rats (Figure 7; one-way ANOVA, F (3, 28) = 16.602, *P* < 0.01). The activity of complex I in the SN was significantly reduced by 26.77% in GDX rats compared to sham rats (*P* < 0.01). Supplements of TP at 1.0 mg/kg, not at 0.5 mg/kg, restored the activity of complex I in the SN of GDX rats to the level in sham rats. No significant differences were detected in the activity of complex II, III, or IV among sham, GDX, GDX-0.5TP, and GDX-1.0TP rats (Table 2).

#### 3.3.3. mtDNA-Encoded Subunits of Complex I

**Figure 8:**
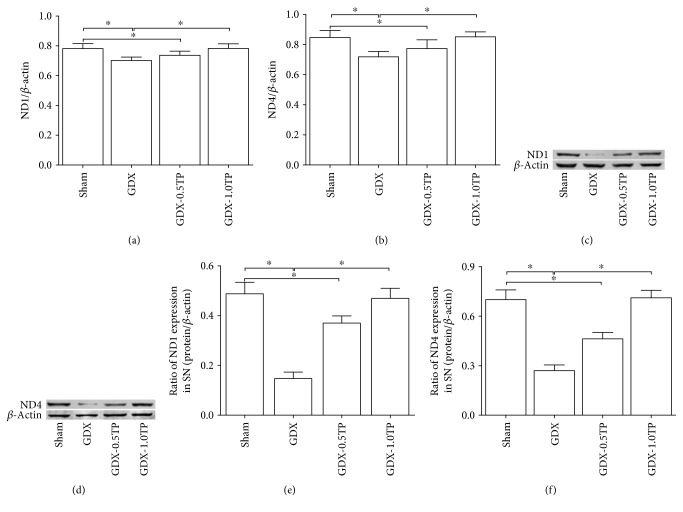
The effects of TP supplements on ND1 and ND4 in the SN of GDX rats. (a-b) ND1 or ND4 mRNA was detected by qPCR. (c–f) ND1 or ND4 protein was measured by Western blot. *n* = 8 per group, expressed as mean ± SD. ^∗^*P* < 0.01.

Of the 7 mtDNA-encoded subunits in complex I, both ND1 and ND4 showed a significant group differences at the level of mRNA among sham, GDX, GDX-0.5TP, and GDX-1.0TP rats (Figure 8(a); ND1: one-way ANOVA, F(3,28) = 14.530, *P* < 0.01; Figure 8(b); ND4: one-way ANOVA, F(3,28) = 16.536, *P* < 0.01). The post hoc test found a 10.23% and 15.24% reduction, respectively, in ND1 mRNA and in ND4 mRNA of GDX rats over sham rats (*P* < 0.01). Supplements of TP to GDX rats at 1.0 mg/kg, not at 0.5 mg/kg, increased ND1 and ND4 mRNAs to the level in sham rats. No significant differences were detected at the mRNA level of ND2, ND3, ND4L, ND5, or ND6 among sham, GDX, and GDX-TP rats (Table 3).

#### 3.3.4. ND1 and ND4

ND1 and ND4 were analyzed at the protein level based on the findings of mtDNA-encoded subunit mRNAs of complex I. Group differences of ND1 and ND4 at the protein level were disclosed in the SN among sham, GDX, GDX-0.5TP, and GDX-1.0TP rats (Figures 8(c) and 8(e); ND1: one-way ANOVA, F(3,28) = 149.744, *P* < 0.01; Figures 8(d) and 8(f); ND4: one-way ANOVA, F(3,28) = 175.603, *P* < 0.01). A 69.85% and 61.44% reduction, respectively, in ND1 protein and in ND4 protein was found in GDX rats over sham rats (*P* < 0.01). The supplements of TP to GDX rats at 1.0 mg/kg, not at 0.5 mg/kg, upregulated ND1 and ND4 to the level in sham rats.

## 4. Discussion

The present study revealed that the testosterone deficiency impaired the mitochondrial function of the substantia nigra and induced the deficits in the nigrostriatal dopaminergic system. The decreased mitochondrial membrane potential, the increased oxidative stress in the SN, and the reduced expression of DA, its metabolites, TH, and DAT in the nigrostriatal dopaminergic system were found in GDX rats. Of the four mitochondrial respiratory chain complexes, castration reduced the activity of mitochondrial complex I in the SN and induced the declined expression of mitochondrial ND1 and ND4 of 7 mtDNA-encoded subunits of complex I. Supplements of TP to GDX rats ameliorated the mitochondrial defects, increased the activity of mitochondrial complex I, and upregulated the expression of mitochondrial ND1 and ND4. The reduced activity of mitochondrial complex I and the downregulated expression of mitochondrial ND1 and ND4 in the SN of 28-day testosterone-deprived rats suggested an important role of testosterone in maintaining the mitochondrial function of the nigrostriatal dopaminergic system and the vulnerability of mitochondrial complex I to testosterone deficiency. Mitochondrial ND1 and ND4, as potential testosterone targets, were implicated in the oxidative damage to the nigrostriatal dopaminergic system.

There is a controversy about androgens and neuroprotection in the central nervous system [6, 43–45]. Dosing of testosterone seems very important in evaluating the results [44]. In the present study, TP supplements to GDX rats at the doses of 0.75, 1.0, and 1.25 mg/kg restored MDA, mitochondrial H_2_O_2_, and mitochondrial GSH/GSSG in the SN to sham levels. However, TP supplements at the dose of 0.5, 1.5, 2.0, 2.5, or 3 mg/kg to GDX rats resulted in infraphysiological or supraphysiological levels of the serum and SN testosterone and induced the increased MDA and mitochondrial H_2_O_2_ in the SN. The results were consistent with the experiments in which moderate, but not very low or very high, doses of testosterone had beneficial effects on behavioral measures such as memory [44], and testosterone at high concentrations induced harmful activity and initiated the apoptosis process [46]. The detected testosterone levels in the serum and SN of GDX rats after TP supplementation at the doses of 0.75, 1.0, and 1.25 mg/kg were in the same levels as those in sham rats, which indicated that the administration of TP at doses of 0.75–1.25 mg to GDX rats reached physiological levels of testosterone.

MDA, an important end product of lipid peroxidation, reflect free radical-mediated cell membrane damage [3, 47]. A marked increase in MDA in the present study suggested the overproduced free radicals and the occurred oxidative stress in the SN of GDX rats. One of the main factors involved in oxidative stress is superoxide anion (O_2_^−^) [48]. Superoxide anion production occurs mainly inside the mitochondrion. It can be converted to O_2_ and H_2_O_2_. GSH and GSSG are redox couples. Glutathione peroxidase catalyzes the reduction of H_2_O_2_ using GSH to produce GSSG and water [49]. The increased H_2_O_2_ and decreased GSH/GSSG in SN mitochondria further revealed the existed oxidative stress in GDX rats. The oxidative stress is closely related to mitochondrial dysfunction [11, 50]. Mitochondrial dysfunction induces free radical overproduction and increases lipid peroxidation [38, 51, 52]. Mitochondria are responsible for generating cellular energy, altering the reduction-oxidation potential of cells, and regulating cell viability [53, 54]. Their function can be evaluated by analyzing the parameters of mitochondria, such as mitochondrial membrane potential [10, 50]. A decrease in the mitochondrial membrane potential can compromise cell viability. The increased lipid peroxidation and mitochondrial H_2_O_2_, as well as the decreased mitochondrial GSH/GSSG and mitochondrial membrane potential in the SN of GDX rats, suggested that testosterone deficiency disturbed the mitochondrial function. Supplements of TP at 1.0 mg/kg to GDX rats restored the mitochondrial membrane potential as well as the status of oxidative stress in the SN. However, TP supplements at 0.5 mg/kg could not restore them in the SN of GDX rats. The present results indicated that the antioxidant role of testosterone [6, 55] might be realized via testosterone-regulating mitochondrial function in some extent. Mitochondrial dysfunction might underlie the defects of the nigrostriatal dopaminergic system of GDX rats. Orchiectomy reduced the expression of DA and its metabolites, as well as that of TH and DAT in the nigrostriatal dopaminergic system. Supplements of TP at physiological levels restored the impaired nigrostriatal dopaminergic system. The restoration by TP supplements of nigrostriatal dopaminergic activity might be involved in the amelioration of behavioral deficits in open-field activity of TP-treated GDX rats. Gonadectomy in male rats decreases the behavioral parameters of open-field activity, such as walking, climbing, and total path length [41]. Supplement of TP improves the decreased behavioral parameters of open-field activity in GDX rats [41].

As an important indicator of mitochondrial function [10, 56], mitochondrial membrane potential is created by the proton gradient across the inner mitochondrial membrane through the proton-extruding system including complexes I, III, and IV of the respiratory chain [10]. Testosterone deficiency might interfere with the mitochondrial function by affecting the proton-extruding system of the mitochondrial respiratory chain. So the influence of TP on complexes I, III, and IV of the respiratory chain in the SN became our main concern in the studies. Of the three mitochondrial respiratory chain complexes in the proton-extruding system, the activity of complex I was altered due to testosterone deficiency. The reduced activity of complex I was found in the SN of GDX rats. The following reasons might explain the reduced activity of complex I in the SN of GDX rats. One is nonspecific oxidative damage. The SN is rich in dopamine. Dopamine itself can be a source of oxidative stress [26], and dopaminergic neurons experience the detriments in auto-oxidation of dopamine [57, 58]. The increased oxidative stress induced by testosterone deficiency might aggravate the oxidative damage of auto-oxidation of dopamine to mitochondrial complexes in the nigrostriatal dopaminergic system of GDX rats. Complex I seem more vulnerable than complexes II, III, and IV to oxidative damage caused by testosterone deficiency. Another reason might be that testosterone specifically regulates the subunits of complex I either via androgen receptor [59, 60] or via estrogen receptor [61, 62] when testosterone is aromatized to estrogen. Whether estrogen was involved in the regulation of complex I subunits is necessary to be investigated in following studies by ovariectomy in female rats. Immunocytochemical [59, 62] and in situ hybridization [60, 61] studies identify the subpopulations of intracellular gonadal hormone receptor-bearing neurons in the SN, which suggested that specific subsets of midbrain dopaminergic neurons might be direct targets of gonadal hormones. Testosterone deficiency specifically caused the decreased activity of complex I and resulted in oxidative stress. The duration after orchiectomy in rats was 28 days. 28-day oxidative stress induced by the reduced activity of complex I in GDX rats might not be enough to affect complexes II, III, and IV. If the duration extended longer, the activities of complexes II, III, and IV would be affected as the ROS was accumulated in GDX rats.

Complex I is a major entry point of the mitochondrial respiratory chain, and its deficiencies constitute an important step in the cascade of events leading to the death of the dopaminergic cells [63]. In 44 subunits of complex I, 7 subunits (ND1, ND2, ND3, ND4, ND4L, ND5, and ND6) are encoded by the mtDNA [64]. In the present study, we found that gonadectomy in male rats resulted in the reduction in ND1 and ND4 of 7 mtDNA-encoded subunits in complex I. Supplements of TP upregulated or restored the expression of ND1 and ND4 in GDX rats, which was dependent on the physiological levels of testosterone in serum and the SN following TP supplementation. Considering the deficits in the nigrostriatal dopaminergic system in GDX rats in the present study and lower testosterone levels in some PD [65, 66] as well as the complex I deficiency in the pathogenesis of PD [16, 18–21], testosterone regulation of both ND1 and ND4 seemed related to the pathogenesis of PD with testosterone deficiency. Manipulating ND1 and ND4 expressions by TP would be a potential approach to prevent mesodopaminergic neurons from further degeneration in testosterone-deficient PD patients in a way. Aging, as an important risk factor for neurodegenerative disorders, is associated with a decrease in protein and mRNA levels of the respiratory chain in mitochondria [15, 38]. Whether testosterone supplements might ameliorate the deficits in mitochondrial protein expression of the mitochondrial respiratory chain in aging should be testified in future studies.

There are some problems unresolved in the present study. We only analyzed MDA, mitochondrial H_2_O_2_, and mitochondrial GSH/GSSG in GDX rats. However, in addition to ROS, oxidative stress has been shown to be associated with reactive nitrogen species (RNS). Thus, the parameters associated with RNS should be measured in GDX rats. Moreover, mitochondrial bioenergetics and ATP production should be detected to confirm the mitochondrial dysfunction. Although 7 mtDNA-encoded transcripts in complex I were mainly analyzed, whether nuclear DNA-encoded complex I mRNAs are also affected in testosterone deficiency is worthwhile further investigation.

In conclusion, testosterone at the physiological levels is necessary for the mitochondrial functions in the substantia nigra. Testosterone deficiency induced the mitochondrial dysfunction and reduced the activity of complex I of the four mitochondrial respiratory chain complexes in the substantia nigra. The reduced activity of complex I was related to the downregulated expression of mitochondrial ND1 and ND4 due to testosterone deficiency. Mitochondrial ND1 and ND4, as potential testosterone targets, were implicated in the oxidative damage to the nigrostriatal dopaminergic system.

## Figures and Tables

**Table 1:** Validation parameters of the LC-MS/MS method.

Analyte	*r*	LLOQ (ng/g)	Recovery (%)	Intraprecision (RSD %)	Interprecision (RSD %)
DA	0.9969	2.0	94.6 ± 8.7	10.3	12.4
DOPAC	0.9982	72.0	93.4 ± 6.6	8.9	7.5
HVA	0.9977	50.0	95.3 ± 7.4	7.7	11.3

DA: dopamine; DOPAC: 3,4-dihydroxyphenylacetic acid; HVA: homovanillic acid.

**Table 2:** Effects of TP on the activity of mitochondrial complexes (*μ*mol/min/g pro).

Enzyme	Sham	GDX	GDX-0.5TP	GDX-1.0TP
Complex I	32.12 ± 3.46	23.52 ± 2.32^∗^	27.96 ± 2.64^∗^^#^	32.21 ± 2.91^#△^
Complex II	21.96 ± 3.08	21.45 ± 2.86	21.69 ± 2.58	22.87 ± 3.41
Complex III	32.72 ± 3.37	31.77 ± 3.78	31.91 ± 3.48	33.06 ± 2.75
Complex IV	15.65 ± 2.02	14.49 ± 2.26	14.55 ± 2.12	16.19 ± 2.22

^∗^ *P* < 0.01 versus sham; ^#^*P* < 0.01 versus GDX group; ^△^*P* < 0.01 versus GDX-0.5TP group.

**Table 3:** Effects of TP on the mRNA levels of mtDNA-encoded subunits of complex I.

Subunits	Sham	GDX	GDX-0.5TP	GDX-1.0TP
ND1	0.78 ± 0.03	0.71 ± 0.04^∗^	0.74 ± 0.03^∗^^#^	0.78 ± 0.04^##△^
ND2	0.85 ± 0.04	0.84 ± 0.03	0.82 ± 0.04	0.86 ± 0.04
ND3	0.93 ± 0.04	0.91 ± 0.03	0.91 ± 0.05	0.92 ± 0.03
ND4	0.85 ± 0.05	0.72 ± 0.03^∗^	0.77 ± 0.06^∗^^#^	0.85 ± 0.03^##△^
ND4L	0.90 ± 0.04	0.89 ± 0.06	0.86 ± 0.06	0.91 ± 0.05
ND5	0.90 ± 0.05	0.88 ± 0.05	0.89 ± 0.05	0.91 ± 0.05
ND6	0.92 ± 0.05	0.90 ± 0.04	0.90 ± 0.05	0.92 ± 0.04

^∗^ *P* < 0.01 versus sham; ^#^*P* < 0.05 versus GDX; ^##^*P* < 0.01 versus GDX; ^△^*P* < 0.01 versus GDX-0.5TP group.
